# Sinapic acid alleviates inflammatory bowel disease (IBD) through localization of tight junction proteins by direct binding to TAK1 and improves intestinal microbiota

**DOI:** 10.3389/fphar.2023.1217111

**Published:** 2023-08-15

**Authors:** Sehyeon Jang, San Kim, Bo Ram So, Younghoon Kim, Chang-Kil Kim, Jeong Jae Lee, Sung Keun Jung

**Affiliations:** ^1^ School of Food Science and Biotechnology, Kyungpook National University, Daegu, Republic of Korea; ^2^ Department of Agricultural Biotechnology, Research Institute of Agriculture and Life Sciences, Seoul National University, Seoul, Republic of Korea; ^3^ Department of Horticultural Science, Kyungpook National University, Daegu, Republic of Korea; ^4^ Institute of Agricultural Science and Technology, Kyungpook National University, Daegu, Republic of Korea; ^5^ Research Institute of Tailored Food Technology, Kyungpook National University, Daegu, Republic of Korea

**Keywords:** sinapic acid, inflammatory bowel disease, early response gene, gut microbiota, transforming growth factor beta-activated kinase 1, activating transcription factor (ATF)-2

## Abstract

**Introduction:** Although sinapic acid is found in various edible plants and has been shown to have anti-inflammatory properties including colitis, its underlying mechanism and effects on the composition of the gut microbiota are largely unknown. We aimed to identify an early response kinase that regulates the localization of tight junction proteins, act at the onset of the inflammatory response, and is regulated by sinapic acid. Additionally, we analyzed the effects of sinapic acid on the homeostasis of the intestinal microbiome.

**Methods:** We examined the aberrant alterations of early response genes such as nuclear factor-kappa B (NF-κB) and activating transcription factor (ATF)-2 within 2 h of sinapic acid treatment in fully differentiated Caco-2 cells with or without lipopolysaccharide and tumor necrosis factor (TNF)-α stimulation. To confirm the effect of sinapic acid on stimulus-induced delocalization of tight junction proteins, including zonula occludens (ZO)-1, occludin, and claudin-2, all tight junction proteins were investigated by analyzing a fraction of membrane and cytosol proteins extracted from Caco-2 cells and mice intestines. Colitis was induced in C57BL/6 mice using 2% dextran sulfate sodium and sinapic acid (2 or 10 mg/kg/day) was administrated for 15 days. Furthermore, the nutraceutical and pharmaceutical activities of sinapic acid for treating inflammatory bowel disease (IBD) evaluated.

**Results:** We confirmed that sinapic acid significantly suppressed the stimulus-induced delocalization of tight junction proteins from the intestinal cell membrane and abnormal intestinal permeability as well as the expression of inflammatory cytokines such as interleukin (IL)-1β and TNF-α *in vitro* and *in vivo*. Sinapic acid was found to bind directly to transforming growth factor beta-activated kinase 1 (TAK1) and inhibit the stimulus-induced activation of NF-κB as well as MAPK/ATF-2 pathways, which in turn regulated the expression of mitogen-activated protein kinase (MLCK). Dietary sinapic acid also alleviated the imbalanced of gut microbiota and symptoms of IBD, evidenced by improvements in the length and morphology of the intestine in mice with colitis.

**Discussion:** These findings indicate that sinapic acid may be an effective nutraceutical and pharmaceutical agent for IBD treatment as it targets TAK1 and inhibits subsequent NF-κB and ATF-2 signaling.

## 1 Introduction

Recent trends such as adoption of westernized diets, irregular meal intakes, and stress result in intestinal diseases such as irritable bowel syndrome, inflammatory bowel disease (IBD), colorectal cancer, and celiac disease (Rizzello et al., 2019). In particular, IBD is a chronic and recurrent disease characterized by inflammation of the intestine. Symptoms of IBD include abdominal pain, diarrhea, bloody stool, weight loss, and weakness (Seyedian et al., 2019). Patients with IBD are at least twice more likely to develop colorectal cancer than the general population, and colorectal cancer caused by IBD is known to be more fatal (Keller et al., 2019).

Once microbial pathogens penetrate to the intestinal barrier and enter lamina propria, they can lead to systemic inflammatory intestinal diseases (Hosomi and Kunisawa, 2020). The maintenance of intestinal barrier requires tight junctions, multiprotein junctional complexes, that selectively control physiological gates that transport ions and solutes (Konig et al., 2016). Tight junctions consist of a complex of transmembrane proteins, such as occludin, claudin, and junctional adhesion molecules and adaptor proteins, such as zonula occludens (ZO)-1 and cingulin (Zihni et al., 2016). Studies on rodents with IBD have revealed that ZO-1 is essential for the formation and maintenance of tight junctions (Zhou et al., 2009). The results of previous study indicate that downregulation of occludin is one of the factors underlying intestinal barrier dysfunction *in vitro* and *in vivo* (Liu et al., 2023).

A diverse signaling network ensures that tight junction proteins between intestinal epithelial cells are properly constructed to maintain gut barrier function. Nuclear factor-kappa B (NF-κB), a transcription factor, plays a central role in inflammation and inflammatory diseases such as IBD, rheumatoid arthritis (RA), multiple sclerosis, atherosclerosis, type I diabetes, and chronic obstructive pulmonary disease (Liu et al., 2020). Once activated NF-κB upregulates the expression of myosin light chain kinase (MLCK) and subsequent phosphorylation of myosin light chain (MLC) in Caco-2 cells (Drolia et al., 2018). A previous study suggested that activating transcription factor (ATF)-2 may serve as an additional transcriptional factor that regulates the expression of *mlck* (Al-Sadi et al., 2013). Although the hyperactivity of ATF-2 is typically associated with inflammatory diseases such as allergic asthma, obesity, and hepatitis (Yu et al., 2014), its role in IBD through the regulation of tight junction protein redistribution via *mlck* expression remains unclear.

Multiple studies have suggested that sinapic acid, found in citrus peels, broccoli, and rapeseed and canola meals, could be a promising nutraceutical and pharmaceutical component that exerts antioxidant (Galano et al., 2011), anti-inflammatory (Wu et al., 2021), and anti-obesity effects (Bae and Kim, 2020). Consumption of natural materials, especially fiber, can also affect the gut microbiota community and protect the intestinal surface as well as alleviate inflammation via the production of short-chain fatty acids (Franzosa et al., 2019). However, the research of natural materials on gut health has primarily focused on the physical function of dietary fibers that improves gastrointestinal motility (Makki et al., 2018). Additionally, the major role of phytochemicals of pharmaceuticals are considered as regulator for pathogenic signaling pathways both in cells and *in vivo*. Interestingly, some recent studies have revealed that polyphenols function as prebiotics through the modulation of the gut microbiome (Xu and Zhang, 2022; Zhang et al., 2023). Therefore, the ability of phytochemicals to act as inhibitors of pathogenic signaling molecules and as prebiotics in the gut, makes them promising gut nutraceuticals for regulating the homeostasis of gut epithelial cells and microbiota.

In this study, we verified the effects of sinapic acid on early response kinase involved in gut inflammation by regulating the localization of tight junction proteins and the gut microbiome in differentiated Caco-2 cells and in mice with DSS-induced colitis. We observed that sinapic acid directly binds to TAK-1 and the subsequent MAPK/ATF-2 and NF-κB signaling pathways. Furthermore, sinapic acid ameliorated DSS-induced colitis and gut microbiota dysbiosis *in vivo*. Based on the above results, we believe that sinapic acid may be an effective nutraceutical or pharmaceuticals agent treating IBD.

## 2 Materials and methods

### 2.1 Material and reagents

Minimum Essential Medium (MEM) with Earle’s Balanced Salts, fetal bovine serum (FBS), nonessential amino acid (NEAA), antibiotics (penicillin/streptomycin solution), and sinapic acid (3,5-dimethoxy-4-hydroxycinnamic acid) were obtained from Thermo Fisher Scientific (Logan, UT, United States). Twelve-well Transwell and Hanks’ Balanced Salt Solution (HBSS) were purchased from Welgene (Gyeongsan, Gyeongsangbuk-do, Republic of Korea). Antibodies against p65, p-p65, IKKα/β, p-IKKα/β, IκBα, p-IκBα, MLCK, p-MLC, E-cadherin, MKK4/7, p-MKK4/7, P38 MAPK, p-P38 MAPK. SAPK/JNK, p-SAPK/JNK, ERK, p-ERK, and α/β-Tubulin were purchased from Cell Signaling Technology (Danvers, MA, United States). Antibodies against lamin B1, ZO-1, occludin, claudin-4, claudin-2, ATF-2, and p-ATF2 were purchased from Abcam (Cambridge, Cambridgeshire, United Kingdom). The primary antibody against β-actin was purchased from Santa Cruz Biotech (Santa Cruz, CA, United States). Lipopolysaccharide (LPS) derived from *Samonella enterica* serotype typhimurium was purchased from Sigma-Aldrich (St. Louis, MO, United States), and tumor necrosis factor (TNF)-α was purchased from NKMAX (Seongnam, Gyeonggi-do, Republic of Korea).

### 2.2 Cell culture and differentiation conditions

Human epithelial colorectal adenocarcinoma Caco-2 cells were purchased from the Korean Cell Line Research Foundation (Seoul, Republic of Korea) and cultured in MEM medium containing 10% FBS, 1% NEAA, and 1% antibiotics (100-U/mL penicillin and 100-μg/mL streptomycin) under a humidified atmosphere of 5% CO_2_ at 37°C. The medium was replenished every 2 days. Caco-2 cells were cultured for 21 days, and to ensure complete differentiation, the MEM medium was replaced every 2 days.

### 2.3 Cell viability

Caco-2 cells were seeded at a density of 1 × 10^6^ cells per mL in 96-well plates, incubated overnight, and subsequently treated with sinapic acid (12.5, 25, and 50 μM) for 24 h. Cell viability was measured using 3-(4,5-Dimethylthiazol-2-yl)-5-(3-carboxymethoxyphenyl)-2-(4-sulfophenyl)-2H-tetrazolium (MTS) and phenazine methosulfate (PMS) reagents (Promega, Madison, WI, United States) according to the manufacturer’s instructions. PMS + MTS solution (1:20) was added (20 μL per well) and incubated for 1 h. The absorbance was measured at 490 nm using a microplate reader (Bio-Rad Inc., Hercules, CA, United States).

### 2.4 Transepithelial electrical resistance (TEER)

Caco-2 cells were seeded at a density of 1 × 10^5^ cells per mL in the apical chambers of 12-Transwell (Corning, NY, United States) and the MEM medium was placed in the basolateral chambers of 12-Transwell. Caco-2 cells were differentiated. After removing the original medium, it was washed twice with HBSS, incubated for 30 min by adding HBSS again, and subsequently stabilized on the bench for 15 min. TEER values were measured using an epithelial volt-ohm meter (Millicell ERS-2, Millipore, Burlington, MA, United States) according to the manufacturer`s instructions at 0 h. Based on our previous study, we confirmed that stimulation with 20 μg/mL LPS and 20 ng/mL TNF-α is optimal for decreasing TEER values. Therefore, in this study, we used a combination of 20 μg/mL LPS and 20 ng/mL TNF-α (hereafter referred to as “stimulus”). Caco-2 cell monolayers were pretreated with 12.5, 25 and 50 μM sinapic acid. After 1 h, the stimulus was administered. TEER values were measured at 0.5, 1, 3, 6, 12, and 24 h following the administration of the stimulus.

### 2.5 Fluorescein isothiocyanate (FITC)-dextran permeability

Caco-2 cells were seeded and differentiated in the same way as the 2.4 TEER measurement method, and sinapic acid and stimulus were treated under the same conditions. After 1 h, 0.5 mL of 1 mg/mL 4 kDa FITC-dextran (Sigma-Aldrich, MO, United States) in HBSS was added to the apical chamber, and 1.5 mL of HBSS was added to the basolateral chamber. After 12 or 24 h, HBSS (100 μL) was drawn from the basolateral chamber and placed on the black bottom plate. The absorbance was measured at 480 nm and 520 nm using SPECTRAmax^®^ GEMINI-XS (Molecular Devices, Sunnyvale, CA, United States).

### 2.6 Quantitative realtime polymerase chain reaction (PCR)

Caco-2 cells were seeded at a density of 1 × 10^5^ cells per mL in the six-well plates. After 1 h of sinapic acid pretreatment, differentiated Caco-2 cells were treated with the stimulus. The total RNA was extracted from Caco-2 cells according to the specifications of RNAiso Plus (Takara Bio Inc., Kyoto, Japan). Next, cDNA was obtained using the ReverTra Ace^®^ qPCR RT Kit & Master Mix (Toyobo, Osaka, Japan) based on the manufacturer’s instructions. The produced cDNA was used for qPCR with SYBR^®^ Green Realtime PCR Master Mix (Toyobo, Osaka, Japan) according to the manufacturer’s instructions. The primer sequences are displayed in Table 1. Relative gene expression levels were normalized using β-actin and the comparative ΔΔCq method using the CFX Maestro Software (Bio-Rad Inc., Hercules, CA, United States).

**TABLE 1:** qPCR primer sequence.

Species	Gene	Sense strand (5′-3′)	Antisense strand (5′-3′)
Human	IL-1β	GCA CAA GGC ACA ACA GGC TGC	CAG GTC CTG GAA GGA GCA CTT CA
	IL-6	TTC TCC ACA AGC GCC TTC GGT CCA	ACG GCT GAG ATG CCG TCG AGG ATG
	TNF-α	ATC AAT CGG CCC GAC TAT CTC	GCA ATG ATC CCA AAG TAG ACC TG
	β-actin	CCT CAC CCT GAA GTA CCC CA	TGC CAG ATT TTC TCC ATG TCG

### 2.7 Drug affinity responsive target stability (DARTS) assay

Caco-2 cells were seeded at a density of 1 × 10^5^ cells per mL in the six-well plates. Differentiated Caco-2 cells were lysed using the M-PER buffer (Thermo Scientific, MA, United States) with a protease and phosphatase inhibitor cocktail (Thermo Scientific, MA, United States). Subsequently, the lysates were centrifuged at 18,000 × g for 10 min at 4°C and supplemented with 10× TNC buffer. After 1 h, the lysates were treated with sinapic acid and digested with pronase (protein-t- pronase ratio, 1:1,000) for 30 min. Digestion was stopped by adding a 5× SDS sample buffer, and proteins were then visualized using western blot assay.

### 2.8 Animal study

Eight-week-old female C57BL/6 mice were purchased from Joongah Bio (Suwon, Republic of Korea). Mice were housed in an air-conditioned room (23°C ± 2) and exposed to a 12/12 h light/dark cycle with free access to food and water. All animals were treated with care, and the study protocol (KNU-2021–0026) was approved by Kyungpook National University and performed in accordance with its animal use guidelines. Mice were randomly allocated into the following groups: control, 2% DSS, 2% DSS +2 mg/kg/day sinapic acid treatment, and 2% DSS +10 mg/kg/day sinapic acid treatment. During the experiment, mice were treated with sinapic acid (2 or 10 mg/kg) daily for 15 days by oral gavage, using an esophageal catheter. Colitis was induced in mice with 2% DSS (MP Biomedicals Korea, Seoul, Republic of Korea). After 15 days, mice were anesthetized with ketamine and sacrificed using the cervical dislocation method.

### 2.9 Membrane/cytosol fraction

For *in vitro* analysis, Caco-2 cells were seeded at a density of 1 × 10^5^ cells per mL in six-well plates for 3 weeks. After 1 h of pretreatment with 50 μM sinapic acid, the stimulus was administered, and cells were incubated for 0.5, 0.75, 1, and 2 h, washed twice with cold PBS, and then centrifuged at 300 ×g for 15 min. For *in vivo* analysis, the mice colon tissues washed with a wash solution were placed in 2 mL microcentrifuge tubes containing 1 mL of cell permeabilization buffer and stainless-steel beads and homogenized using Precellys 24 dual tissue homogenizer (Bertin, Montigny-le-Bretonneux, Montigny-le-Bretonneux, France). Subsequently, 500 μL cell permeabilization buffer was added to homogenized tissues. Homogenized tissues were centrifuged at 16,000 ×g for 15 min. The separation of cytoplasmic and membrane proteins of obtained cells and homogenized colon tissues was performed using membrane solubilization buffer and cell permeabilization buffer (Thermo Scientific, Logan, UT, United States) according to the manufacturer’s protocol. Separated proteins were then visualized using a western blot assay.

### 2.10 Nucleus/cytosol fraction

Caco-2 cells were seeded at a density of 1 × 10^5^ cells per mL on six-well plates. Differentiated cells were pretreated with 50 μM sinapic acid for 1 h and then treated with the stimulus. Cells were washed twice with cold PBS. Caco-2 cells were scraped and collected in 1 mL PBS and then each sample was centrifugated at 500 ×g for 5 min. Following PBS removal, cytoplasmic and nuclear proteins were separated using NE-PER Nuclear and Cytoplasmic Extraction Reagents (Thermo Scientific, Logan, UT, United States) according to the manufacturer’s protocol. Separated proteins were then visualized using western blot assay (Kim et al., 2022).

### 2.11 Western blot assay

For *in vitro* analysis, Caco-2 cells were seeded at a density of 1 × 10^5^ cells per mL on six-well plates. Differentiated cells were pretreated with 50 μM sinapic acid for 1 h and then treated with the stimulus. Cells were washed twice with cold PBS. Caco-2 cells were scraped and collected in 200 μL of lysis buffer (Cell Signaling Technology, Danvers, MA, United States) containing a protease and phosphatase inhibitor cocktail (Thermo Scientific, MA, United States). For *in vivo* analysis, mice colon tissues washed with PBS were placed in 2 mL microcentrifuge tubes containing 500 μL of lysis buffer and stainless-steel beads and homogenized using Precellys 24 dual tissue homogenizer. Homogenized tissues were centrifuged at 13,652 ×g for 15 min. Lysed cells and colon tissues were vortexed and maintained on ice for 30 min and subsequently centrifuged at 18,928×g for 15 min. The total protein content was quantified using the DC Protein Assay Kit reader (Bio-Rad Inc., Hercules, CA, United States) and separated by 8%, 10%, and 12% SDS-PAGE gels. The total protein was transferred from the gel to 0.45 μm PVDF membranes (Immobilon-P transfer membrane, Millipore, Burlington, United States), blocked with 5% skim milk in Tris Buffered Saline with Tween ^®^ 20 (TBST) buffer for 1 h at room temperature, and then hybridized with primary antibodies overnight at 4°C. Following hybridization with horseradish peroxidase (HRP)-conjugated secondary antibody (Thermo Scientific, MA, United States) for 1 h at room temperature, immune-reactive proteins were detected using a chemiluminescence detection kit (ATTO, New York, United States) and GeneGnome XRQ NPC (Syngene, Nuffield Rd, United Kingdom).

### 2.12 Immunofluorescence analysis

For *in vitro* immunofluorescence analysis, Caco-2 cells were seeded at a density of 1 × 10^5^ cells per mL on an eight-well chamber (ibidi, GmbH, Gräfelfing, Germany) for 3 weeks. Cells were pretreated with 25 and 50 μM sinapic acid for 1 h and then treated with the stimulus. For *in vivo* immunofluorescence analysis, colon tissues were fixed using an OCT compound (Leica Microsystems, Seoul, Republic of Korea) and cryosectioned into 10 μM sections. Cells and sectioned colon tissues were fixed with 4% formaldehyde for 15 min and washed three times with PBS. Cells and sections were permeabilized with ice-cold 100% methanol and then blocked and incubated overnight at 4°C with the primary antibodies occludin (1:100) and ZO-1 (1:200). After washing, the cells and sections were incubated with a goat anti-rabbit immunoglobulin G H&L conjugated to the Alexa Fluor^®^ 488 and 647 secondary antibodies (Abcam, Cambridge, United Kingdom) for 1 h. Subsequently the cell nucleus was stained with VECTASHIELD^®^ Antifade Mounting Medium with DAPI (Vector Laboratories Inc., Burlingame, United States).

### 2.13 Hematoxylin and eosin (H&E) staining and histology analysis

Colon tissue sections were stained with filtered 2.9% hematoxylin (Sigma-Aldrich, St. Louis, MO, United States). After rinsing in cool running water for 5 min, sections were dipped in 0.5% eosin (Sigma-Aldrich, St. Louis, MO, United States) five times. After rinsing in water for 5 min, sections were sequentially dehydrated using 50% ethanol, 70% ethanol, and 95% ethanol. Sections were then dipped in xylene (Sigma-Aldrich, St. Louis, MO, United States) for cleaning and mounted with quick-hardening mounting medium (Sigma-Aldrich, St. Louis, MO, United States) for observation. The histological evaluation of colon injury was conducted by three investigators in a blind setting. The criteria for assigning histological evaluation scores of colon injury are described in Table 2 (Fung and Putoczki, 2018).

**TABLE 2:** Colonic damage criteria for histology analysis.

Score	Epithelial damage	Mucosal inflammation	Submucosal *i*nflammation
0	Normal	None	None
1	Hyper-proliferative	Mild	Mild
2	<50% crypt loss	Moderate	Moderate
3	>50% crypt loss	Severe	Severe
4	100% crypt loss		
5	Ulceration		

### 2.14 Microbiota analysis

DNA was extracted from mice fecal samples using the Powerfood Microbial DNA Isolation kit (Mo Bio Laboratories, Inc., Carlsbad, CA, United States) according to the manufacturer’s protocol. Intestinal bacterial community compositions were analyzed using 16S rRNA gene sequencing, which was performed according to a previous report (Ryu et al., 2022). The obtained data were analyzed using Mothur (v. 1.45.3). To visualize differences in the microbiota composition between groups, nonmetric multidimensional scaling plots were generated on the basis of unweighted and weighted UniFrac distances. The α-diversity and relative abundance of OTUs at family and genus levels were analyzed using the MicrobiomeAnalyst web-based platform (Chong et al., 2020).

### 2.15 Statistical analysis

Data were expressed as mean ± standard deviations of three independent experiments wherever appropriate. Significant differences were calculated using one-way analysis of variance and Tukey’s *post hoc* multiple comparison test. A *p*-value of <0.05 was considered statistically significant.

## 3 Result

### 3.1 Sinapic acid inhibits stimulus-induced dysfunction of intestinal permeability and cellular redistribution of tight junction proteins in differentiated Caco-2 cells

**FIGURE 1:**
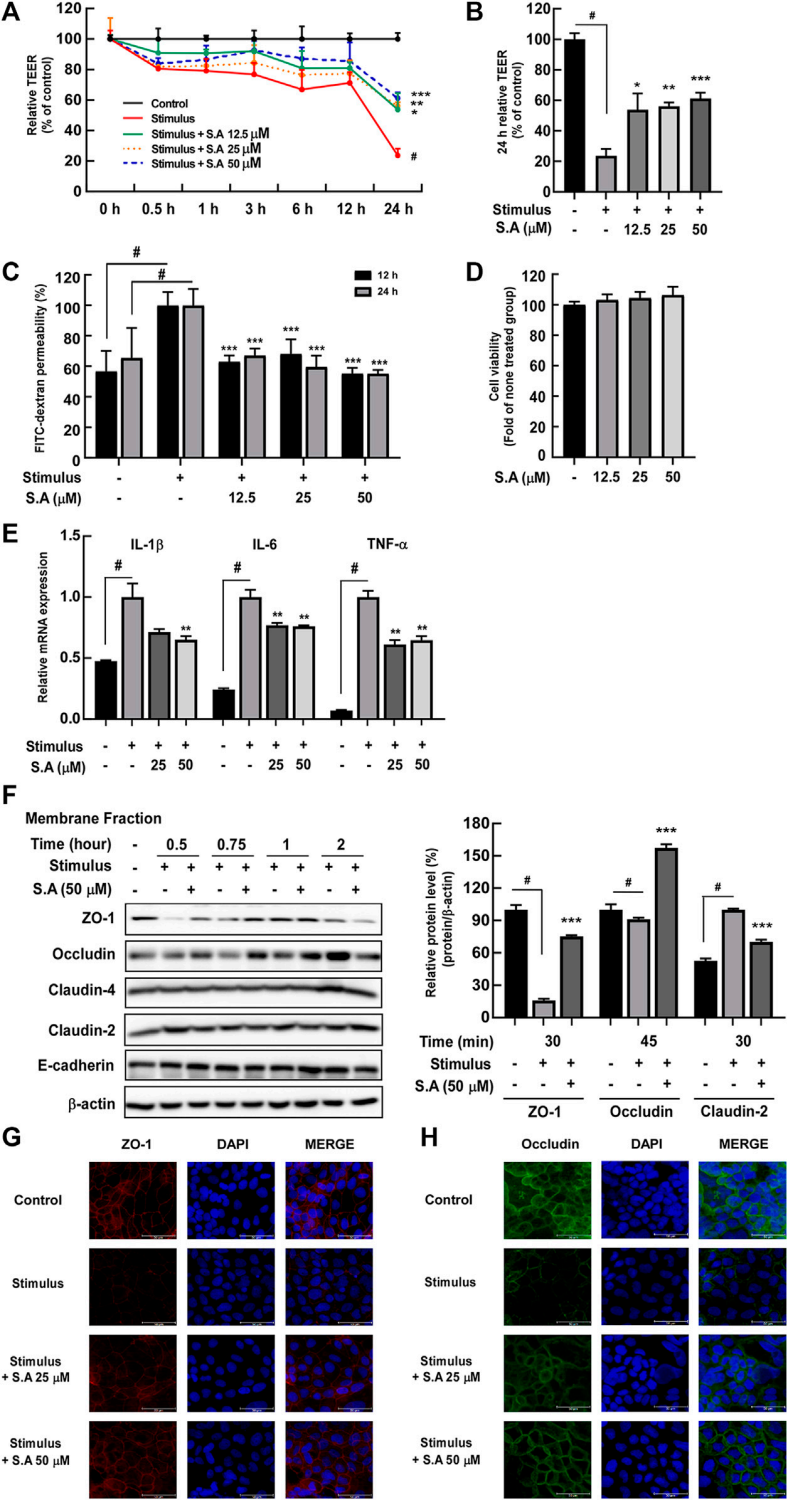
Effects of sinapic acid on stimulus-induced epithelial permeability and inflammatory cytokine levels in Caco-2 cells. Sinapic acid inhibits stimulus-induced TEER values **(A,B)** at 24 h and FITC–dextran permeability **(C)** in Caco-2 cells. **(D)** Cell viability. **(E)** Sinapic acid inhibits the stimulus-induced expression of IL-1β, TNF- α, and IL-6 in Caco-2 cells. **(F–H)** Sinapic acid inhibits the stimulus-induced localization of ZO-1, claudin-2, and occludin in Caco-2 cells. Data are presented as means ± standard deviations (SDs) (*n* = 3). #*p* < 0.05 versus the control group. *p < 0.05, **p < 0.01, and ***p < 0.001 versus the stimulus group.

To verify the nutraceutical and pharmaceutical activities of sinapic acid in the treatment of IBD, we evaluated TEER value and FITC as the major indicators for determining intestinal permeability *in vitro*. The results demonstrated that sinapic acid significantly inhibited stimulus-induced decrease in the TEER value (Figures 1A, B) and FITC-dextran permeability at both 12 and 24 h (Figure 1C) without inducing cell cytotoxicity in Caco-2 cells (Figure 1D). Additionally, sinapic acid significantly suppressed the stimulus-induced mRNA expression of proinflammatory cytokines IL-1β, IL-6, and TNF-α in differentiated Caco-2 cells (Figure 1E). The proper localization of tight junction and adherence junction proteins in the membranes of intestinal epithelial cells plays a critical role in regulating intestinal permeability and maintaining the integrity of the intestinal epithelial barrier (Odenwald and Turner, 2017). Therefore, we analyzed the changes in the localization of tight junction proteins induced by sinapic acid over time. After fractionating the membrane proteins and conducting western blot assays, we found that sinapic acid significantly suppressed the stimulus-induced delocalization of ZO-1 and claudin-2 at 30 min and occludin at 45 min in the membrane fraction of Caco-2 cells (Figure 1F). Immunofluorescence analysis photographs also support these results (Figures 1G, H). Based on these results, we confirmed that sinapic acid regulates abnormal intestinal barrier permeability within a relatively short period, (i.e., 30 and 45 min) by inhibiting the delocalization of tight junction proteins in cell membranes under inflammatory conditions.

### 3.2 Sinapic acid inhibits stimulus-induced MLCK/MLC/NF-κB signaling pathways and phosphorylation of ATF-2 in differentiated Caco-2 cells

**FIGURE 2:**
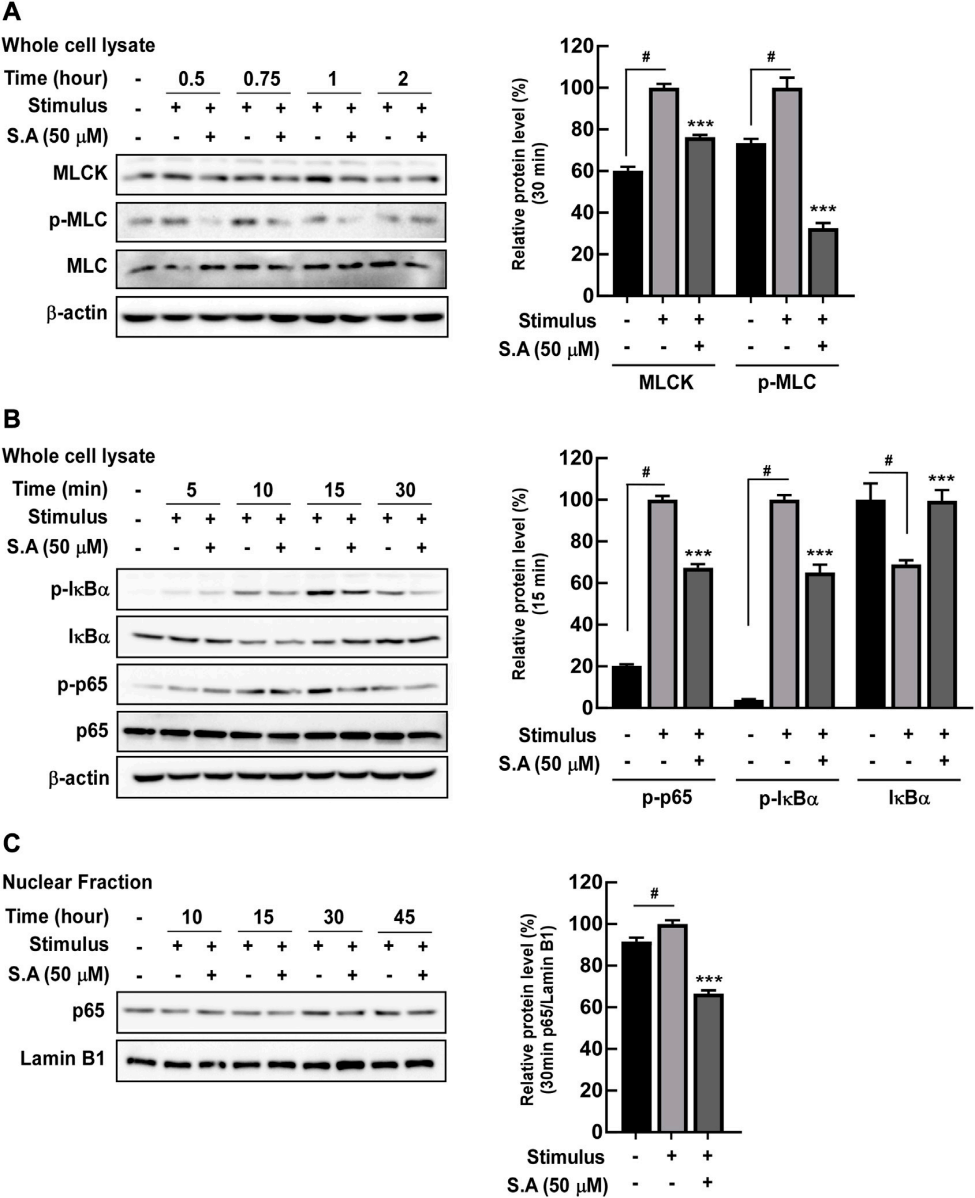
Effects of sinapic acid on the stimulus-induced expression of MLCK, phosphorylation of MLC, and NF-κB pathway activation in Caco-2 cells. Sinapic acid inhibits stimulus-induced MLCK/p-MLC **(A)** and IκBα and p65 phosphorylation **(B)** as well as p65 translocation in the nuclear fraction from Caco-2 cells **(C)**. **(A,B)** MLCK, p-IκBα and IκBα are quantified relative to β-actin, while p-MLC and p-p65 are quantified relative to their whole forms. Data are presented as means ± SDs (*n* = 3). #*p* < 0.05 versus the control group. *p < 0.05, **p < 0.01, and ***p < 0.001 versus the stimulus group.

**FIGURE 3:**
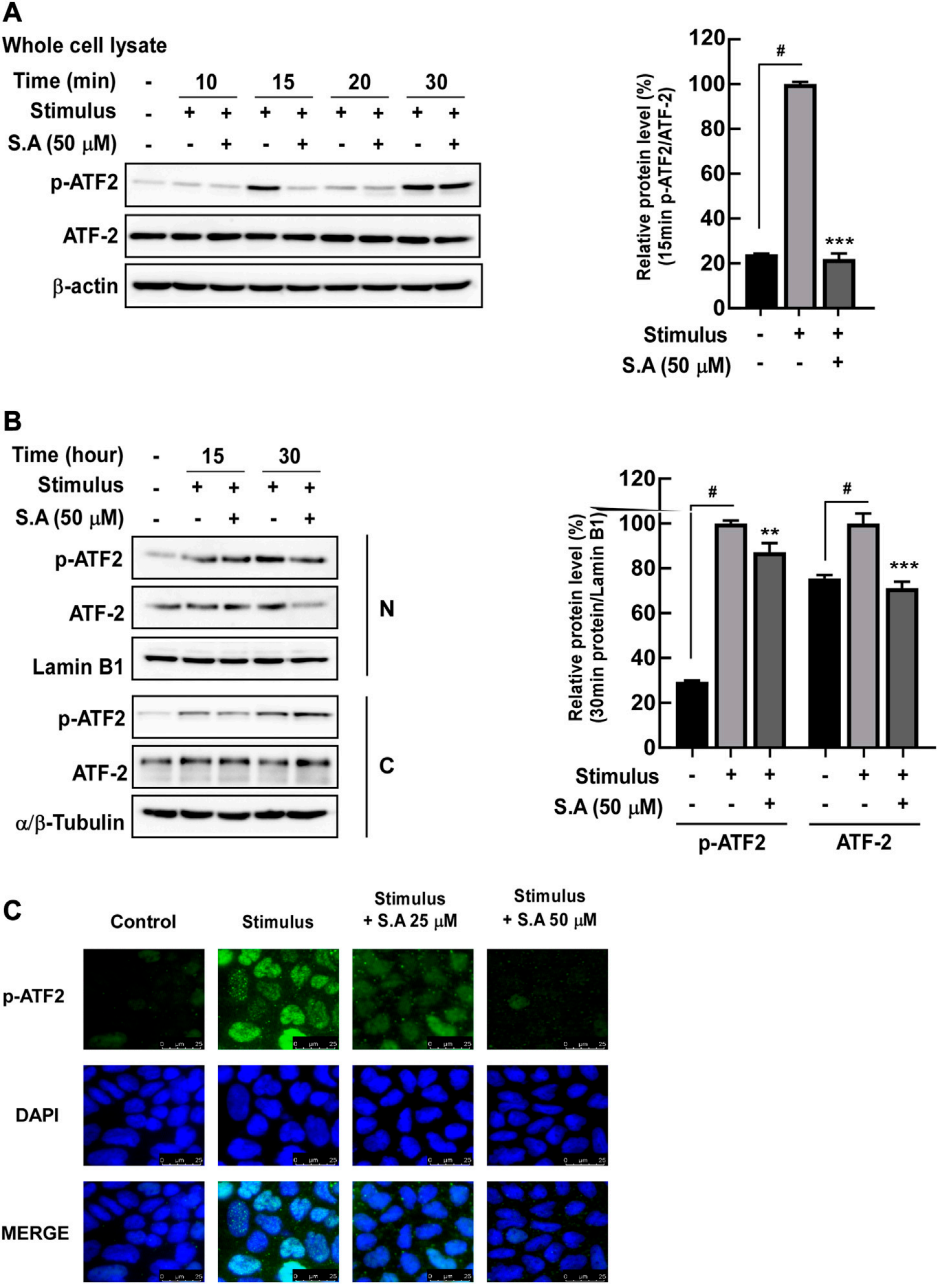
Effects of sinapic acid on the stimulus-induced phosphorylation of ATF-2. **(A)** Sinapic acid strongly inhibits stimulus-induced phosphorylation of ATF-2 at 15 min in Caco-2 cells. **(B,C)** Sinapic acid inhibits stimulus-induced translocation of ATF-2 in nuclear and cytosolic fractions from Caco-2 cells. Data are presented as means ± SDs (*n* = 3). #*p* < 0.05 versus the control group. *p < 0.05, **p < 0.01, and ***p < 0.001 versus the stimulus group.

Owing to the rapid effect of the stimulus on the delocalization of tight junction proteins, we focused on the impact of sinapic acid on early response inflammatory signaling molecules including NF-κB. NF-κB is a transcriptional factor for *mlck*, which subsequently phosphorylates MLC, leading to the delocalization of tight junction proteins (Drolia et al., 2018). We found that sinapic acid inhibited stimulus-induced increase in MLCK expression and phosphorylation of MLC at 30 min (Figure 2A) and phosphorylation of p65 and IκBα at 15 min (Figure 2B). Lan et al. reported that sinapic acid suppressed the stimulus-induced translocation of p65 at 30 min by about 30% compared to the stimulus only group (Figure 2C). A recent study`s demonstrated that ATF-2 is a novel transcription factor for *mlck* expression (Kaminsky et al., 2021). In addition to NF-κB, we found that ATF-2, another transcriptional factor that regulates *mlck*, was phosphorylated at 15 min and translocated to the nucleus at 30 min following stimulus administration (Figures 3A, B). These alternations were suppressed by sinapic acid (Figures 3A–C).

### 3.3 Sinapic acid inhibits stimulus-induced phosphorylation of MAPKs and directly binds to TAK1 in differentiated Caco-2 cells

**FIGURE 4:**
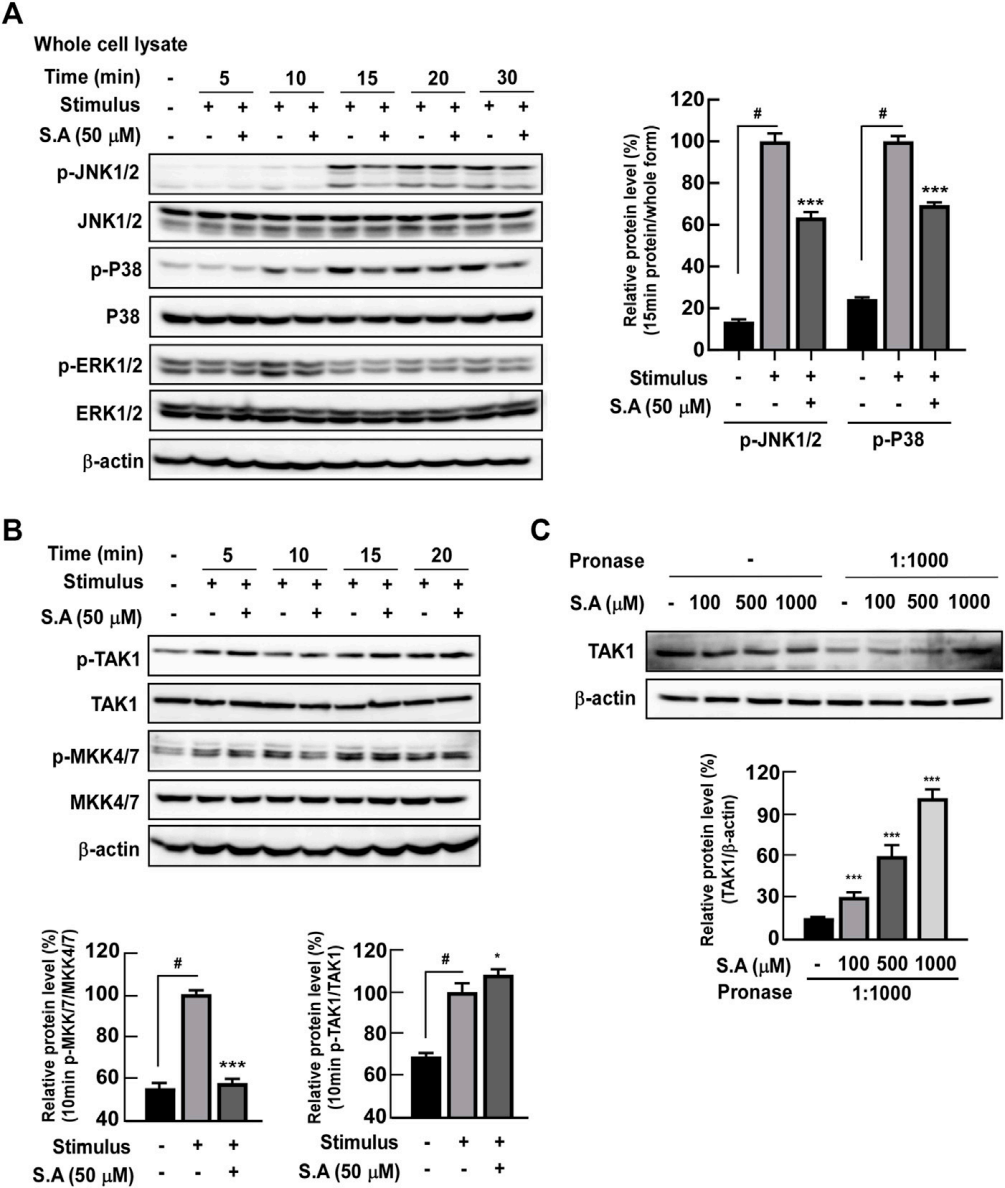
Effects of the sinapic acid binding ability to TAK-1 and subsequent MAPK signaling pathways in Caco-2 cells. **(A)** Sinapic acid inhibits the stimulus-induced phosphorylation of JNK1/2 and P38 in Caco-2 cells. **(B)** Sinapic acid prevents stimulus-induced phosphorylation of MKK4/7 in Caco-2 cell but not TAK1in Caco-2 cells. **(C)** Sinapic acid directly binds to TAK-1 in Caco-2 cells. Data are presented as means ± SDs (*n* = 3). #*p* < 0.05 versus the control group. *p < 0.05, **p < 0.01, and ***p < 0.001 versus the stimulus group.

In our previous study, we have shown that phytochemicals directly target early response kinases and subsequent inflammation and carcinogenesis *in vitro* and *in vivo* (Jung et al., 2008). Therefore, we hypothesized that sinapic acid may affect upstream kinase that regulates NF-κB and ATF-2 transcriptional factors. Phosphorylation of MAPK was confirmed under the hypothesis that MAPK may be involved in the phosphorylation of ATF-2 (Desai et al., 2013). Sinapic acid significantly inhibited the stimulus-induced phosphorylation of JNK1/2 and p38 at 15 min of stimulus exposure in differentiated Caco-2 cells but did not affect the phosphorylation of ERK1/2 (Figure 4A). Sinapic acid inhibited the stimulus-induced phosphorylation of MKK4/7 at 10 min in differentiated Caco-2 cells (Figure 4B). Although we initially suspected that TAK1 could be a major regulator of MAKK and NF-κB pathways, we found that sinapic acid did not affect the phosphorylation of TAK1 (Figure 4B). Therefore, we predicted that sinapic acid may directly affect TAK1 by binding to it. The DARTS assay revealed that sinapic acid suppressed pronase-induced TAK1 degradation in differentiated Caco-2 cells (Figure 4C). These results collectively suggest that sinapic acid directly bound to TAK1, which suppressed the activation of TAK1-mediated NF-κB and ATF-2 transcriptional factor.

### 3.4 Oral administration of sinapic acid alleviates DSS-induced IBD and modifies the gut microbiota

**FIGURE 5:**
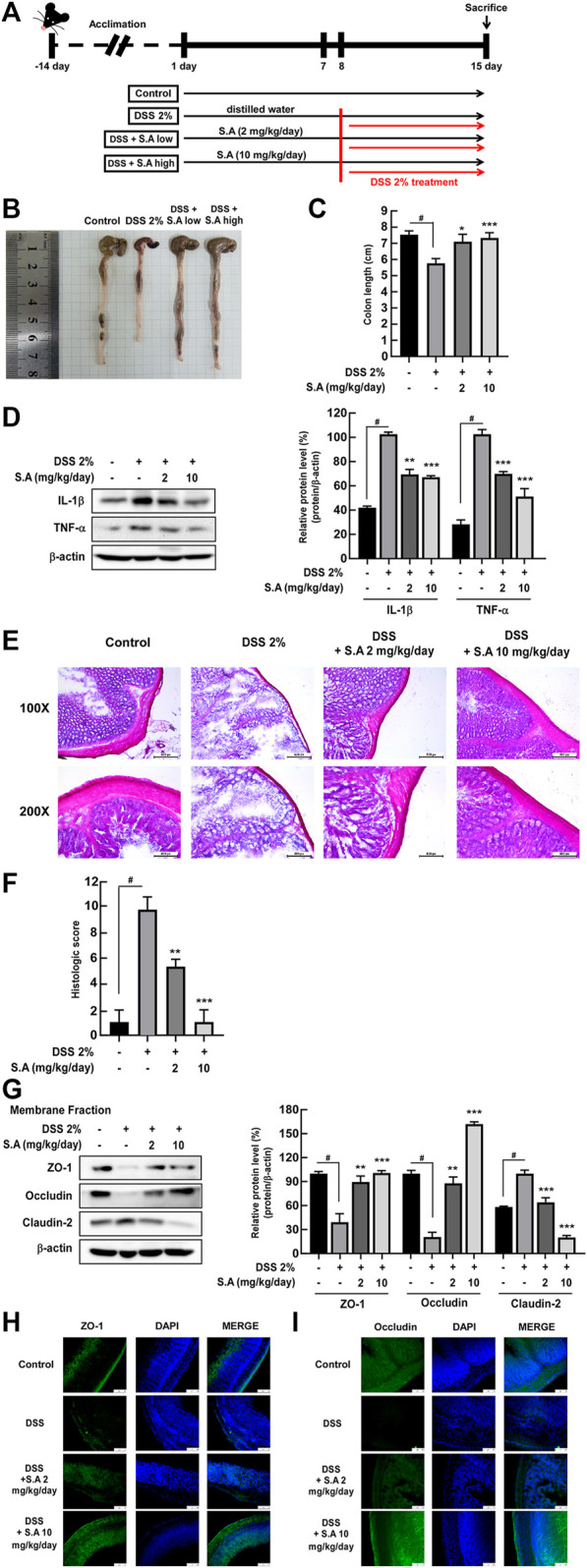
Effects of sinapic acid on DSS-induced inflammatory bowel disease *in vivo*. **(A)** Schematics of the DSS-induced colitis model. The oral administration of sinapic acid inhibits DSS-induced colon shortening **(B,C)** production of cytokines, including TNF-α and IL-1β **(D)**, loss of crypts and mucosal layer destruction in the colon tissues **(E)**, increase in histological scores **(F)** and localization of ZO-1, occludin, and claudin-2 in the membrane fraction of the colon tissues **(G–I)**. #*p* < 0.05 versus the control group, *p < 0.05, **p < 0.01, and ***p < 0.001 versus the 2% DSS group.

Although studies have shown that sinapic acid alleviates colitis in TNBS-induced Balb/c female mice (Lee, 2018) and DSS-induced Kunming mice (Qian et al., 2020), the effect of sinapic acid on the homeostasis of gut microbiota has not yet been elucidated. Sinapic acid was administered orally for 15 days from 8 days before DSS intake, and ulcerative colitis was induced via *ad libitum* intake of 2% DSS for 8 days (Figure 5A). We observed that oral administration of sinapic acid ameliorated DSS-induced colon shortening (Figures 5B, C) and production of inflammatory cytokines, including IL-1β and TNF-α (Figure 5D) in mice. H&E staining and histological scores of mice colon tissue indicated that oral administration of sinapic acid mitigated DSS-induced colon tissue damage, such as loss of crypts and destruction of the mucus layer (Figures 5E, F). Western blot assay using a membrane fraction of mice colon, along with immunofluorescence analysis, showed that sinapic acid effectively suppressed the DSS-induced delocalization of ZO-1, occluding, and claudin-2 (Figures 5G–I). These results confirmed that the *in vitro* localization of tight junction proteins was also reflected in animal intestinal tissues.

**FIGURE 6:**
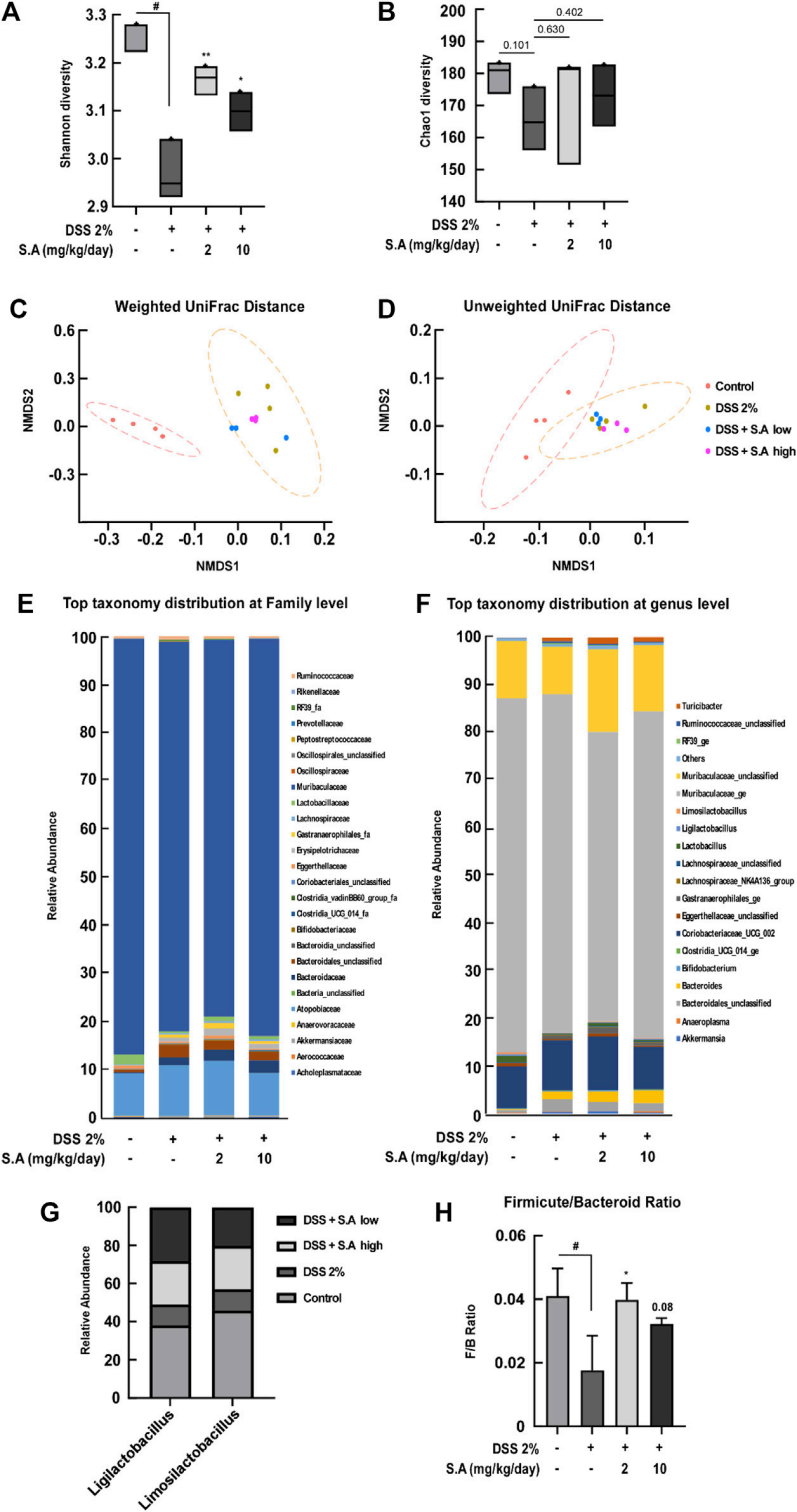
Effects of sinapic acid on the DSS-induced composition of the gut microbiota. **(A)** Shannon diversity and **(B)** Chao1 diversity. **(C)** Weighted UniFrac distance and **(D)** Unweighted UniFrac distance. **(E)** Relative abundance of the family level. **(F)** Relative abundance of the genus level. **(G)** At the genus level, an abundance of *Ligilactobacillus* and *Limosilactobacillus* in the feces of C57BL/6J mice is observed. **(H)** The Firmicutes-to-Bacteroidetes ratio in the feces of C57BL/6J mice. Data are presented as means ± SDs (*n* = 3). #*p* < 0.05 versus the control group, *p < 0.05 and **p < 0.01 versus the 2% DSS group.

A recent study has summarized current findings that phytochemicals may act as prebiotics for modulating the gut microbiota (Xu and Zhang, 2022). However, current nutraceutical research on gut health is focused on dietary fiber that improves gastrointestinal motility (Makki et al., 2018). Therefore, we aimed to evaluate the effect of oral administration of sinapic acid on changes in intestinal microflora. Shannon diversity, an indicator of microbial richness and evenness, and Chao1 diversity, an indicator microbial richness, are representative α-diversity indexes. Oral administration of sinapic acid significantly prevented DSS-induced decrease in Shannon diversity (Figure 6A). Although there was no significant difference, sinapic acid prevented DSS-induced decrease in Chao1 diversity (Figure 6B). Next, we examined β-diversity distances using weighted and unweighted UniFrac distances. Weighted and unweighted UniFrac distances significantly differed between the control and 2% DSS groups (*p* < 0.001; Figures 6C, D). Although not significant, the weighted UniFrac distance from sinapic acid groups to the control group was smaller than the distance from the 2% DSS group to control group (Figure 6C). There was no significant difference in the composition of microbiota at the family level between the groups (Figure 6E). However, at the genus level, we found that sinapic acid inhibited DSS-induced declines in the relative abundance of *Ligilactobacillus* and *Limosilactobacillus* in feces of C57BL/6J mice (Figures 6F, G). *Limosilactobacillus reuteri* exerts anti-inflammatory effects, ameliorates hepatic disorders, and prevents infections caused by enterohemorrhagic *Escherichia coli* and *Helicobacter pylori* through the production of bacteriocins (Abuqwider et al., 2022). Firmicutes and Bacteroidetes are the major phyla of human gut microbiota, and the Firmicutes to Bacteroidetes ratio (F/B ratio) has been widely studied as an indicator of the correlation between gut microbes and intestinal health (Stojanov et al., 2020). Oral administration of sinapic acid was found to suppress DSS-induced decrease in F/B ratio (Figure 6H). These results demonstrate, for the first time, that sinapic acid can act as a prebiotic.

## 4 Discussion

Despite the role of intestine in digesting food, absorbing nutrients, and inhabiting intestinal microorganisms, dietary habits and social environment can pose a threat to the intestinal health. Individuals recognize that an unbalanced and irregular diet, stress, and exposure to environmental pollution threaten intestinal health; however, there is a limit to changing them simultaneously. Therefore, taking nutraceuticals and pharmaceuticals is a highly efficient and accessible way for maintaining intestinal health for individuals living a busy life in today’s modern world. Multiple lines of study have reported that phytochemicals can be a promising nutraceutical and pharmaceutical for gut health (Hossen et al., 2020). Interestingly, a recent study has suggested that phytochemicals, like fibers, act as prebiotics, modulate the composition of the gut microbiota, and maintain intestinal integrity (Martel et al., 2020). Therefore, in this study, we focused on the development of nutraceutical and/or pharmaceutical phytochemicals capable of both pathogenic inhibition and prebiotic ability.

To verify the candidate of nutraceutical and pharmaceuticals of sinapic acid in IBD treatment, we evaluated TEER values and FITC as the major indicators for determining intestinal permeability *in vitro*. In our previous study, we established the optimal conditions for measuring intestinal permeability in fully differentiated Caco-2 cells using a combination of 20 ng/mL TNF-α and 20 μg/mL LPS (So et al., 2023); this condition was labeled as the “stimulus” and used in all subsequent *in vitro* experiments. Multiple assay results showed that sinapic acid has a preventive effect on gut epithelial permeability with an increase in TEER values and a decrease in FITC-dextran permeability by approximately 38% and 45%, respectively, at nontoxic concentrations. The maintenance of intestinal epithelial integrity is strongly associated with tight junctions and adherent junctions that formed between intestinal epithelial cells (Konig et al., 2016). A previous study has demonstrated that sinapic acid affects the expression of tight junction proteins at 24 h in Caco-2 cells (Lan et al., 2021). However, in our study, inflammation conditions (LPS and/or TNF-α) did not affect tight junction protein levels within 24 h of stimulus exposure in differentiated Caco-2 cells (So et al., 2023). Therefore, we belived that there are other mechanisms of sinapic acid to sustain gut homeostasis. Following the fractionation of membrane and cytosolic tight junction proteins, we confirmed that sinapic acid mitigated the stimulus-induced redistribution of ZO-1 and occludin from the membrane to the cytosol in differentiated Caco-2 cells. In Figure 1F, we observed a notable trend that the sinapic acid-treated group had higher distribution of occludin in membrane fraction compared to the untreated group. Further research is needed to explore this phenomenon comprehensively and gain a deeper understanding of the underlying mechanisms. Drolia et al. proved that the redistribution of tight junction proteins, including occludin and claudin-1, is the critical cause of intestinal epithelial barrier dysfunction by adhesion and translocation of *Listeria* (Drolia et al., 2018). Therefore, the inflammation of intestine strongly pulls tight junction proteins from the membrane to the cytosol, thereby resulting in intestinal epithelial barrier dysfuctions; sinapic acid seems to play a role in maintaining the localization of ZO-1 and occludin to the membrane rather than its expression.

Although several studies showed that sinapic acid alleviates TNBS-induced (Lee, 2018) and acetic acid (Shahid et al., 2022) in rodent-colitis models, its target molecules in gut inflammation are majorly unknown. It is well known that NF-κB has a central role in the development of inflammation and inflammatory metabolic diseases including obesity, type 2 diabetes, and atherosclerosis (Baker et al., 2011). In IBD, activated NF-κB upregulated MLCK expression/phosphorylation and subsequently increased the delocalization of tight junction proteins, including ZO-1 and claudin, from the membrane of intestinal epithelial cells, thereby leading to intestinal barrier permeability (Konig et al., 2016). Although sinapic acid significantly suppressed the MLCK/p-MLC signaling network increased by the stimulus, p65 phosphorylation and translocation to the nucleus inhibited it by 30% and 35%, respectively, which was relatively low. As a transcription factor that regulates the *mlck* gene expression, the activated ATF-2 by phosphorylated p38 binds to the *mlck* promoter and is consequently involved in intestinal barrier permeability in differentiated Caco-2 cells (Al-Sadi et al., 2013). Moreover, we confirmed that sinapic acid suppressed the stimulus-induced MKK4/7/JNK1/2 and p38 phosphorylation and the subsequent ATF-2 phosphorylation and translocation to the nucleus by 70% and 30%, respectively. With these observations, we believed that ATF-2 is one of the significant factors for modulating the *mlck* gene expression, and these signaling pathways are susceptible to sinapic acid.

Interestingly, phosphorylation of TAK1, an upstream kinase that regulates MKK4/7 phosphorylation, was not affected by sinapic acid. In our previous studies, we reported that the compounds do not affect the kinase itself but substrates or downstream molecules are regulated by those direct binding in skin carcinogenesis (Jung et al., 2008; Ha et al., 2018) and drug-resistant lung cancer (Jung et al., 2014). Therefore, we hypothesized that sinapic acid directly binds to TAK1 and downregulates its downstream signaling molecules, including MKK4/7/JNK1/2 and p38. Drug affinity response target stability (Lomenick et al., 2009) and western blot assay results support our hypothesis that sinapic acid directly binds to TAK1 and suppresses stimulus-induced phosphorylation of early response kinase, MKK4/7/JNK1/2 and p38-ATF2 and ATF2 translocation from the cytosol to the nucleus. Therefore, the interaction between sinapic acid and TAK1 blocks ATF-2 and the subsequent MLCK-p-MLC–mediated redistribution of tight junction proteins, thereby leading to intestinal barrier permeability. If the specific binding site for sinapic on TAK1 is confirmed by conducting co-crystallography analysis of the refined TAK1 and sinapic acid, it can provide that more specific information on the safety and mechanism of action of sinapic acid as a new nutraceutical and phytomedicine.

To evaluate whether the intestinal barrier protection effect of sinapic acid in the *in vitro* experiment was reflected in the biological system and the effect on the improvement of intestinal microflora, an *in vivo* experiment was performed. We used a 10 mg/kg/day dose of sinapic acid as the maximum concentration in the DSS-induced colitis in a C57BL/6 female mice model. Others used DSS-induced colitis in Kunming mice were administered with 50 mg/kg of sinapic acid (Qian et al., 2020). As the major symptoms of IBD, colon length shortening, colon tissue damage, and increased inflammatory cytokines including IL-1β, TNF-α, were significantly suppressed by the oral administration of sinapic acid. *In vitro* experiment results demonstrated that the oral administration of sinapic acid inhibits the redistribution of tight junction proteins by DSS through the analysis of mouse intestinal membrane fractionated proteins.

Dysbiosis, which is the transition in the composition of intestinal microbiota, leads to various intestinal diseases. In particular, a decrease in the Firmicutes group is mainly observed in patients with IBD (Stojanov et al., 2020). The gut microbiota can be remodeled by the phenolic compound resveratrol (Chen et al., 2016; Zhang et al., 2019; Zhang et al., 2020), phlorizin mainly found in apple root (Zhang et al., 2019), and alliin present in garlic (Zhang et al., 2020). These studies suggest that phytochemicals may act as prebiotics for modulating the gut microbiota. However, current nutraceutical research on gut health is focused on dietary fibers that improve gastrointestinal motility (Makki et al., 2018; Gill et al., 2021). Therefore, novel nutraceutical studies that alleviate intestinal permeability and modulate the gut microbiota are needed. We confirmed that dietary sinapic acid prevents DSS-induced imbalance of the gut microbiota, similar to the control level. Specifically, the Firmicutes to Bacteroidetes ratio, which is decreased in inflammatory diseases, increased more in the 2% DSS + sinapic acid group than in the 2% DSS group. Dietary sinapic acid significantly mitigated the DSS-induced decrease in the abundance of *Ligilactobacillus* and *Limosilactobacillus* belonging to the Firmicutes phylum. *Ligilactobacillus* and *Limosilactobacillus*, which are mainly observed in vertebrates are attracting attention as probiotics. *Limosilactobacillus reuteri* produces effects, including anti-inflammatory, mitigation of hepatic disorders, and prevention of *Enterohemorrhagic E. coli* and *H. pylori* infection, through the production of bacteriocin (Abuqwider et al., 2022). Clinical studies showed that *Ligilactobacillus salivarius* improves allergic diseases, including asthma (Drago et al., 2022). For the first time, these results demonstrated that sinapic acid can act as a prebiotic. Future studies are warranted to study the effect of sinapic acid on human-derived intestinal microbes using gnotobiotic mice, as study will increase the possibility of its application as prebiotics.

We confirmed that sinapic acid directly bound to TAK1 and subsequently suppressed the phosphorylation of early response molecules including NF-κB and ATF-2 within 30 min of stimulus exposure. This consequently contributed to the localization of tight junction proteins, including ZO-1, claudin-2, and occludin, within 45 min of stimulus exposure in fully differentiated Caco-2 cells. Additionally, the oral administration of sinapic acid exhibited a suppressive effect on DSS-induced colitis in mice and sustained the diversity of intestinal microflora reduced by DSS, which indicates that sinapic acid has a combined activity of disease-causing gene inhibitor and prebiotics. Therefore, we believe that sinapic acid could be a promising nutraceutical and pharmaceutical agent for the treatment of IBD.

Abbreviations: ATF-2, activating transcription factor-2; DSS, dextran sulfate sodium; IBD, inflammatory bowel disease; IL-1β, interleukin-1β; LPS, lipopolysaccharide; MAPK, mitogen-activated protein kinase; MLC, myosin light chain; MLCK, myosin light chain kinase; NF-κB, nuclear factor-kappa B; TAK1, transforming growth factor beta-activated kinase 1; TNF-α, tumor necrosis factor-α; ZO-1, zonula occludens-1.

## Data Availability

The original contributions presented in the study are included in the article/Supplementary Material, further inquiries can be directed to the corresponding authors.
